# Informal employment, population health, and welfare policies: A global empirical analysis between 2011–2021

**DOI:** 10.1371/journal.pone.0325277

**Published:** 2025-06-26

**Authors:** Amanda E. Aronsson, Indra de Soysa

**Affiliations:** 1 Centre for Global Health Inequalities Research (CHAIN), Department of Sociology and Political Science, Norwegian University of Science and Technology (NTNU), Trondheim, Norway; 2 Department of Sociology and Political Science, Norwegian University of Science and Technology, Trondheim, Norway; University of Maribor Faculty of Arts: Univerza V Mariboru Filozofska Fakulta, SLOVENIA

## Abstract

Despite informal employment being the most common type of employment globally, the empirical link between informality and health is underexplored. Using time-series, cross sectional data from the International Labor Organization (ILO) on informal employment for roughly 126 countries between 2011–2021, this study applies OLS regression to explore how informality associates with population health, measured as healthy life expectancy (HALE), maternal mortality, under-five mortality, equality of access to health care, and mortality due to communicable diseases and maternal and prenatal undernutrition. We also address whether this relationship is conditioned by the availability of higher quality welfare state. The results show that informality associates with a host of measures of poor health, controlling for important confounders, but it associates weakly positively with HALE. Contrary to expectations, an accessible welfare state does not condition informality in ways that lower the health burden. Our results are possibly not causal but subject to endogeneity bias since bad health conditions could lead to increased welfare policies. Longer time series of informal employment data is needed for further assessment. The basic results, however, point to a connection between the size of the informal workforce and diminished population health, controlling for important confounders.

## Introduction

Most of the global workforce, roughly 61%, are informally employed, meaning that they are not subject to national labour legislation, income taxation, employment-based social protection, and unemployment benefits [1]. Informal employment is most prevalent in low- and middle-income countries (LMICs). In these countries, informal workers are, for example, day labourers, street vendors, waste pickers, or home-based workers such as garment makers [1]. In high-income countries (HIC), the term ‘undeclared work’ taking place in the ‘shadow economy’ [2] is commonly used to refer to a similar phenomenon, namely unprotected and unregistered employment. Informal workers can be employers, employees, self-employed or contributing family-members [1], and what defines informality within these heterogeneous groups is that formal rules do not regulate this type of worker and they are denied employment-based protections.

Because informal work falls outside the scope of labour market regulation and legislation, informal employment is also often highly precarious. Precarious employment as a multidimensional concept is a measure of the (poor) quality of employment. It covers the three dimensions of income inadequacy, employment insecurity and a lack of rights and protections [3]. Informal employment is a type of employment arrangement that, by definition, is characterized by employment insecurity and a lack of rights and protections, and informal employment is thus usually to some degree precarious. While informal and precarious employment are not necessarily the same thing, they often overlaps, which means that research on the latter, can be informative also for questions concerning informal employment.

In theory, informal employment could have direct health effects, although it is more likely that health effects occur through factors located at the micro-, meso- and macro-levels [4,5]. At the micro-level, material factors, social factors, and working conditions, influence the link between informality and health. For example, poor psychosocial working environment, and stress resulting from employment insecurity and low income, may cause poor mental health. At the meso-level, workplace- and sector-specific factors are considered. Finally, institutional factors associated with the labour market, such as labour regulations, and the nature of welfare policies might influence the links between informality and health at the macro-level. Many of these pathways remain to be explored empirically.

Given that more than half of the world’s working population works informally, informal employment as a health determinant has not received sufficient attention within the public health literature. Indeed, relatively few studies appear in in existing reviews on this topic [6,7]. A better understanding of informal employment as a health determinant is important also in relation to reaching the Sustainable Development Goals, SDGs, for example SDG 8 to promote sustained, inclusive and sustainable economic growth, full and productive employment and decent work for all, and target 8.3 which specifically refers to informal employment. However, scientific evidence that informal employment associates with adverse health outcomes and health inequalities, is increasing. For example, informal workers often report adverse general and mental health compared to formal workers [8,9], and women tend to be more severely affected by informality compared with men, illustrated by the stronger and more frequently reported statistically significant relationships between informally working women and adverse health effects [10–12]. Women and children are often the most vulnerable members of society. Informality and precarious employment can also affect the health and well-being of other family members [13–15], and has been linked to the mortality levels of general populations [16]. While informality thus likely impacts health beyond individual workers, this potential spillover effect is largely neglected. Work and employment are known to be important determinants of health [17], thus with most workers globally being informally employed, links to the health of the general population needs to be better understood in relation to this specific form of employment. Therefore, the remainder of this paper addresses how the size of the informal workforce could be linked to population health in general.

By focusing on the size of informality in a country, we assume that the spillover effects of informality on other household-members and the community matter. There are three basic assumptions underlying this argument. Firstly, that the strains often experienced by informal workers, such as stress, low income or poor mental health, may influence the health and wellbeing of family-members in the same way as has been seen for other precarious work [15,18,19]. Thus, the larger the informal workforce, the greater the share of the general population that is exposed to the consequences of informality manifested in population health.

Secondly, the larger the informal workforce, the smaller the taxable population, which means fewer resources for governments to invest in health-benefitting services and other interventions [20], such as education, vaccination programmes, social policies and other health infrastructure. Furthermore, since taxation often leads to representation, the non-taxed informal sector likely has little political influence, reducing representation and increasing such a group’s irrelevance to government agencies. Thus, people who fall outside a state’s taxable income (informal workers) will likely be neglected by being excluded from state services, increasing health inequalities, and lowering overall population health standards. Communicable diseases spread if a large portion of people remain unvaccinated, for example.

Finally, institutional factors, especially welfare policies, could be important at the macro-level, influencing the links between informality and population health. As informal workers are denied employment-based protections, family-members are directly influenced by the vulnerability of such workers. For example, the lack of parental leave can have health effects on children as mothers, who are still carrying the main burden of childcare responsibility globally, may face challenges to provide safe childcare or to breastfeed while still having to work [21]. Studies based on individual-level data show that women’s and children’s health indicators, for example nutritional status and antenatal care utilization, often is more adversely affected by informal employment [7,10,22]. This effect is likely partly due to their particular vulnerability to the lack of social protections. The interplay between access to welfare policies and the size of the informal workforce should thus be linked to population health outcomes involving women and children, such as maternal and child mortality. Furthermore, the lack of sick leave or income protections may force workers to attend work while bearing an illness, thus exposing others to the disease, because they fall outside the scope of labour protections and welfare protections. For example, the implemented protection packages during Covid-19 generally did not reach informal workers, leading to severe loss of income that otherwise could go to health benefitting purposes for them and their families. This resulted in many informal workers continuing to work, exposing others around them to Covid-19 [23].

The lack of attention to health effects of the general population in existing research on informal employment is accompanied by insufficient attention to pathways [4]. Despite the recognition of the potential moderating effects of macro-level factors on the health effects of employment in general [5,24], with some exceptions [16,25–27], few studies assess how the relationship between informality and health is moderated by institutional factors. It may be that the health consequences of informality are conditioned by governance mechanisms that smooth the vulnerabilities of informal workers. In the words of Esping-Andersen [28], the ways in which governments “decommodify” market relations have consequences for economic and social life. The moderating role of labour market regulations and welfare states on population health, thus needs greater attention.

A common approach to explore how macro-level welfare state factors influence the links between employment and health, is to consider the moderating role of welfare state *regimes*. This approach has been applied to studies of general employment conditions mainly in HICs [29], although a handful of studies on informal employment have been conducted in the Latin American and Central American regions. The underlying theoretical assumptions are similar in these studies despite institutional heterogeneity, namely that more universal and generous welfare state regimes make workers more resilient to external shocks and sudden life events, which ultimately protects health and reduces health inequalities [26,30]. Thus, an accessible system might be thought of as a better “quality” system because coverage matters for preventing disease. Yet, empirical evidence on the welfare state as a moderating factor between employment conditions, health and health inequalities is mixed. In HIC-settings, the hypothesis that more universal and generous welfare states (such as the Scandinavian welfare state regimes) moderate the effects of precarious employment is supported by some studies [31]. Still, others refute them [30]. Similar mixed trends have been found when applied to health inequalities between formal and informal workers [16,26,27]

The inconclusive results on the role of welfare states, indicate that there is more room for investigating conditional effects between informality and the quality of welfare states for assessing the relationships, particularly using broader samples of countries and for a broad range of health outcomes. Yet, a regime framework is not the only relevant approach to analyse the role of the welfare state on health inequalities [32]. The *institutional* approach considers the effects of welfare states often by focusing on the design of existing policies, and the *expenditure* approach assesses the effects of welfare states by public spending on welfare and social policy [32,33]. Indeed, it may not be the type of regime that matters but the actual targeted nature of social protections. Because governments in larger informal economies should have less revenues to spend on welfare than governments of smaller informal economies, the expenditure approach might be a poor fit for comparative research. Instead, an institutional approach fits this purpose better, where welfare states are assessed, not in general common characteristics (regime approach) or their size in allocated revenues (expenditure approach), but according to the design of social policies that allow universal access. The policy design determines eligibility, revealing the quality of access and thus the nature of potential outcomes. As some suggest, scholars should pay much greater attention to institutional designs of welfare provision and their relative effects on health outcomes [33].

Finally, because women and children are often most vulnerable to the potential consequences of informality [7] and given the importance of social policies for making this group more resilient to poverty and ill-health [34], attention to the health of these groups as a result of the interplay between informality and welfare policies is needed [35]. Thus, the current literature presents a research gap on how informal employment affects not only the workers themselves, but how it is linked to population health outcomes in the wider society. There is also a need to explore how macro-level factors interacts with informality and health, such as welfare policies. And finally, health outcomes specifically relevant for women and children should more keenly examined. The rest of the article assesses theory on informality and health, presents the aims of the empirical contribution of this paper, discusses the data and methods, discuses results, and briefly concludes.

### Aims and identification strategy

The aims of this paper are to examine empirically whether and to what extent informal employment, across a wide range of countries and over multiple years, is linked to population health outcomes, and then to assess if the health effects of informality are conditioned by access to higher quality welfare state policies. We examine the effects across a range of health outcomes, specifically health outcomes identified as mattering for women and children. We hypothesize that informality is inversely associated with population health and that this inverse relationship is conditional on access to higher quality welfare policies. We also assume that informality affect the health of men, women and children differently, which could be detected especially prominently in health measured on indicators salient to women and children, such as maternal mortality and under-five mortality rates.

Our identification strategy is to assess the association between health and the size of informality. Correlation is not causation. We suggest that an association, although not necessarily causally linked, tells us something about the direction and strength of a relationship identified by plausible mechanisms examined above. While a population´s overall health status is affected by many factors, we believe a strong relationship would support the view that employment conditions matter, above the important confounders controlled in the models. If the mechanisms we identified above do not hold, then the association between x and y and its strength would reveal the plausibility of the explanation.

### Data and method

#### Independent variable.

Our independent variable, informal employment, comes from a time-series cross-sectional (TSCS) dataset where each country (between 120–126) is observed annually from 2011–2021. The data are unbalanced depending on data availability on each unit’s variables over time across the panels. Informal employment data is obtained from the ILO Labour Statistics [36] and consists of harmonized microdata from national household surveys of the working-age population (15–64 years). The operational definitions to identify informal employment in the national datasets have been clearly defined [37], allowing for comparisons across countries over time. We divide the number of informally employed workers by total population to measure the potential “size of informality” within a given country.

We estimate models for the female informal worker share of the population, which correlates with the total share of informal workers at r = 0.96, which suggests that male and female numbers are similar across countries (see intercorrelation matrix in S1 Appendix). We also correlate our measure of informal employment with two alternative estimates of the size of the informal economy relative to GDP used widely by economists [38,39]. These estimates are derived from complex modelling of various macro- and micro-economic, political and market conditions, and household decisions under which informality is expected to increase. Our measure of the size of informal employment correlates at r = 0.67 with Schneider´s and r = 0.72 with Elgin and Oztunali´s measures of the size of the shadow economy. Our measure, thus, associates strongly with allied concepts of informality even if differently measured and thus shows a high degree of validity and reliability.

#### Outcomes variables.

We use a variety of variables to measure population health. First, we use the health adjusted life expectancy (HALE) sourced from the GBD 2019 data [40]. HALE is a multi-dimensional vector of health, measured as health-adjusted life expectancy, which is developed to map the global burden of disease from the GBD data on mortality and morbidity. HALE subtracts years of life lost to disease (YLL) and/or years of life with disability (DALY) from simple life expectancy based on years lived. YLL and DALY are based on 369 diseases plus injuries – from violence, self-harm, transport and occupational accidents, and unhealthy lifestyle choices like consumption of drugs, tobacco, and alcohol. It is a catch-all indicator, and like all catch-all indicators is hard to unpack. The HALE index also has other problems: many observations for many of the poorest countries are estimates derived from complex modelling exercises using local-level air and water pollution, economic growth rates, etc. This may bias results particularly for the poorest countries, where data availability and quality are problematic [41]. More importantly, the disease burden in lower-income countries and higher-income countries is also likely to be for very different reasons given that lower-income countries mostly suffer communicable diseases [42]. In contrast, the higher-income countries suffer non-communicable ones driven by lifestyle-based health issues [43]. This means their disease burdens are ‘caused’ by two different sets of problems—poverty versus relative wealth. The GBD researchers have striven to rectify the worst imperfections; thus, we believe that HALE is still a valid, reliable measure of overall population health that captures the aggregate state of public health and deserves addressing [43].

We also use measures of population health that are more narrowly defined, focusing specifically on maternal and child health. We test the under-five mortality rate, the maternal mortality rate, and mortality due to communicable diseases and maternal and prenatal nutritional conditions. These data are sourced from the World Development Indicators (WDI) online database [44]. These measures should supplement our findings based on the broader HALE measure and allow us to explore how informality specifically affects the health of the groups most vulnerable to informality: women and children [35]. The correlations between HALE and these other narrower indicators of mortality are very high (see intercorrelation matrix in appendix).

Our final outcome of interest is health inequalities measured in terms of access to good health. We use an expert coded indicator of the equal access to health taken from the Varieties of democracy project [45]. Expert coders are then questioned on the degree to which governments provide equal access to health between rich and poor. These coding are then subject to rigorous analysis based on Item Response Theory for increasing reliability [45,46]. We obtain a correlation of r = −0.76 between equal access to health and the under-five mortality rate of the WDI data, which suggests high correspondence between objective data in terms of the rate of child death and subjectively coded data based on expert opinion about the equality of access to healthcare.

#### The moderating variable.

To test the conditional effects of whether the quality of welfare state policies moderate informality´s effects on health, we interact our informality measure with an indicator of the quality of a welfare state, taken from the VDEM project. Following the reasoning of the VDEM coders, we suggest that a high-quality welfare state is designed as universally accessible rather than designed as means-tested. The presence of a universal welfare state signals less polarization between social groups and that a state desires to protect all citizens regardless of their socio-economic positions in society. A means-tested system might be easily manipulated for political exigencies, and many people are likely to be excluded for clietalistic reasons. A universal welfare state is distinguishable from a means-tested system in the following manner. According to the VDEM coders:

A means-tested program targets poor, needy, or otherwise underprivileged constituents. Cash-transfer programs are normally means-tested. A universal (non-means tested) program potentially benefits everyone. This includes free education, national health care schemes, and retirement programs. Granted, some may benefit more than others from these programs (e.g., when people with higher salaries get higher unemployment benefits). The key point is that practically everyone is a beneficiary, or potential beneficiary. The purpose of this question is not to gauge the size of the welfare state but rather its quality [45].

The VDEM variable of a universal welfare state used here, does not measure true access to social protections since access to social policies, even in a universal welfare state, often is highly linked to formal employment.

#### Control variables.

We control for a number of broad factors that reduce spuriousness of the findings linking informality with health. We keep to a few controls to allow cleaner interpretation of the results of our main variable of interest [47]. First, we control for GDP per capita. Income level measured by GDP per capita tells us something about the expected size of informality, and it predicts population health status [42,48]. We obtain a high correlation (r = −0.75) between GDP per capita and informality, suggesting that informality is generally well explained by the GDP of a country. The GDP per capita data are taken from the WDI and represent income in constant 2015 USD. Secondly, we control for the level of democracy (political governance). The level of democracy should affect the shape and form of informality in society, and democracy should directly affect population health due to higher provision of public goods [49,50]. We use the basic (minimal) definition of democracy of the VDEM project, which is electoral democracy, where higher values signify that elections are competitive and free, with no coercion or electoral violence [51]. Thirdly, we directly control for serious political upheaval and violence by accounting for whether a civil war involving the state and a rebel group(s), where at least 25 battle-related deaths have occurred in a single year [52]. This discrete variable takes the value 1 if a civil war is ongoing and 0 if not. These data are sourced from the Uppsala Conflict Data Project [52,53]. Additionally, from the same data source, we compute the number of years a country has enjoyed peace since the last civil war, or the year 1946. Finally, we add a time trend to capture mutually trending effects between our x and y variables (time fixed effects).

### Statistical analysis

Our estimating method is pooled OLS regression using clustered standard errors that are robust to heteroscedasticity and serial correlation, treating the within-unit observations as non-independent but independent across [54]. Analyses were conducted using STATA version 18. A full correlation matrix and summary statistics of each of our variables appear in the S1 Appendix and the S2 Appendix. We initially conduct basic additive regressions to examine how informality associates with the various population health outcomes, followed by the inclusion of conditional effects between informality and the quality of a welfare state. We add two conditional terms to the basic regression equation 1) the size of informality * a quality welfare state, where the dependent variables will be HALE and 2) the same interaction with the under-five mortality rates as the dependent variable. These conditional terms are best assessed with margins plots showing the effect of x1 on y at each level of x2, considering the significance level of each effect along the scale at the 95% confidence interval (Braumoeller, 2004). We use the method (interflex) that relaxes the assumption that the conditional terms have a linear effect and model potential non-linear dynamics in the conditional effects [55].

## Results

Table 1 column 1 reports results of the additive models testing the share of informal workers on HALE.

**Table 1:** OLS regressions of the share of informal workers on various population health outcomes, 2011-2021.

Dependent variables	DV1	DV2	DV3	DV4	DV5
Informal workers/population (ln)	0.01	0.26***	0.46***	0.32***	−0.30***
	(0.01)	(0.06)	(0.11)	(0.11)	(0.11)
GDP per capita (ln)	0.06***	−0.52***	−0.74***	−0.33***	0.49***
	(0.01)	(0.06)	(0.09)	(0.08)	(0.11)
Electoral democracy	−0.04	0.03	0.79	1.16**	0.59
	(0.03)	(0.28)	(0.52)	(0.45)	(0.64)
Civil war ongoing	−0.01	0.21	0.10	0.32	−0.06
	(0.02)	(0.17)	(0.29)	(0.28)	(0.30)
Civil peace experience (years)	−0.00	−0.00	−0.01	−0.01	0.01**
	(0.00)	(0.00)	(0.01)	(0.00)	(0.01)
Constant	3.61***	6.64***	8.64***	3.73***	−3.45***
	(0.06)	(0.52)	(0.88)	(0.98)	(0.99)
Observations	642	704	480	633	763
R-squared	0.640	0.811	0.755	0.476	0.660

Note: DV1 = HALE. DV2 = Under-five mortality rate. DV3 = Maternal mortality rate. DV4 = Mortality due to communicable diseases and maternal and prenatal nutritional conditions.DV5 = Equal access to health. Robust standard errors in parentheses.

*** p < 0.01, ** p < 0.05, * p < 0.1.

year fixed effects estimated.

standard errors clustered on country.

As seen there, the effect of informality is positive but statistically not different from zero. Except for per capita GDP, none of the controls matter. As expected, per capita GDPs effect is positive and statistically significantly associated with higher health-adjusted life expectancy. Richer countries have better health. In column 2, however, when we test the under-five mortality rate, informality shows the expected positive effect, which is statistically highly significant. Substantively, holding each of the controls at their mean values, a standard deviation increase in the size of informality, increases the under-five mortality rate by 27% of a standard deviation, which amounts to roughly 6.2 children per 1000 live births (the substantive effects are calculated as the standardized coefficient of X using the formula ((BetaX*sdX)/sdY)*100). In column 3, again, informality associates positively with the maternal mortality ratio. A standard deviation increase in informality increases maternal mortality by 34% of a standard deviation, amounting to 48.4 additional deaths of mothers linked to childbirth. These results are independent of per capita GDP and the rest of the controls.

In column 4, we test the effects of informality on mortality due to communicable diseases and maternal and prenatal nutritional conditions. Again, the size of informality predicts higher levels of death from nutritional conditions and communicable diseases, an effect that is statistically highly significant. Substantively, a standard deviation increase in informality increases deaths due to communicable diseases and nutrition-related causes, such as stunting, by 36% of a standard deviation of the share of deaths attributable to communicable disease and undernutrition-related causes. These results too are independent of the controls. As seen in column 5, again as expected, higher informality associates negatively with equal access to health, a result that is statistically highly significant. Substantively, a standard deviation increase in informality reduces equal access to health by roughly 23% of a standard deviation of equal access to health.

In Table 2, we test only the share of the female informal workers on the same dependent variables as Table 1. As seen there the results are similar. This is not surprising given the high correlation between men’s and women’s share in informal work.

**Table 2:** OLS regressions of the share of female informal workers on various population health outcomes, 2011-2021.

Dependent variables	DV1	DV2	DV3	DV4	DV5
Female informal workers/population (ln)	0.01	0.24***	0.46***	0.32***	−0.26**
	(0.01)	(0.06)	(0.10)	(0.10)	(0.11)
GDP per capita (ln)	0.06***	−0.52***	−0.73***	−0.31***	0.50***
	(0.01)	(0.06)	(0.09)	(0.08)	(0.12)
Electoral democracy	−0.05	−0.12	0.55	1.03**	0.77
	(0.03)	(0.30)	(0.52)	(0.44)	(0.66)
Civil war ongoing	−0.01	0.21	0.14	0.35	−0.07
	(0.02)	(0.18)	(0.29)	(0.29)	(0.30)
Civil peace experience (years)	−0.00	−0.00	−0.01	−0.01	0.01**
	(0.00)	(0.00)	(0.01)	(0.00)	(0.01)
Constant	3.65***	7.02***	9.11***	3.97***	−4.00***
	(0.05)	(0.51)	(0.82)	(0.88)	(0.90)
Observations	642	704	480	633	763
R-squared	0.636	0.809	0.757	0.481	0.656

Note: DV1 = HALE. DV2 = Under-five mortality rate. DV3 = Maternal mortality rate. DV4 = Mortality due to communicable diseases and maternal and prenatal nutritional conditions.DV5 = Equal access to health. Robust standard errors in parentheses.

*** p < 0.01, ** p < 0.05, * p < 0.1.

year fixed effects estimated.

standard errors clustered on country.

Next, in Table 3, we examine the basic model with the additive effects of having a universal welfare state on population health plus the conditional effects of informality and a higher quality welfare state for predicting HALE and under five mortality rates. In Table 3, columns 1 and 2, we test the additive effects on each dependent variable.

**Table 3:** Additive and conditional effects of informality and access to a universal welfare state on population health.

Dependent variables	DV1	DV2	DV13	(2
Universal welfare state	0.01	−0.05	−0.00	−0.02
	(0.01)	(0.06)	(0.01)	(0.12)
Informal workers/ population (ln)	0.01*	0.25***	0.01	0.27***
	(0.01)	(0.06)	(0.01)	(0.08)
GDP per capita (ln)	0.06***	−0.52***	0.06***	−0.52***
	(0.01)	(0.06)	(0.01)	(0.06)
Electoral democracy	−0.05	0.10	−0.05	0.11
	(0.03)	(0.29)	(0.03)	(0.29)
Civil war ongoing	−0.01	0.20	−0.01	0.19
	(0.02)	(0.17)	(0.02)	(0.17)
Peace exposure (years)	−0.00	−0.00	−0.00	−0.00
	(0.00)	(0.00)	(0.00)	(0.00)
Informal workers/pop * Welfare state			0.00	−0.01
			(0.01)	(0.04)
Constant	3.61***	6.68***	3.61***	6.64***
	(0.06)	(0.52)	(0.06)	(0.53)
Observations	642	704	642	704
R-squared	0.642	0.812	0.643	0.812

Note: DV1 = HALE. DV2 = Under-five mortality rate. Robust standard errors in parentheses.

*** p < 0.01, ** p < 0.05, * p < 0.1.

year fixed effects estimated.

standard errors clustered on country.

**Fig 1:**
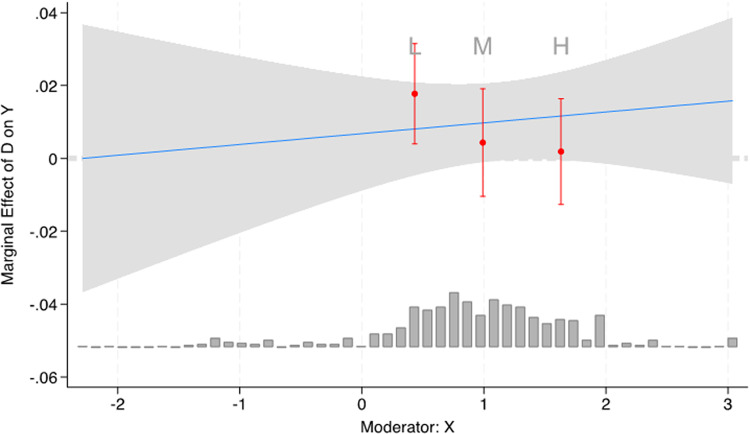
Margins plot of conditional effects of informality and access to welfare state on HALE.

As seen there, there is no statistically significant effect of a universal welfare state on either of the two dependent variables. The share of informal workers is now positive and statistically significant at the 10% level for HALE and positive and highly significant when testing the under-five mortality rates. At least for this sample of countries, for these years, a higher percentage of informal workers increases health adjusted life expectancy. The conditional effects in columns 3 and 4 are best viewed as margins plots because the significance levels of X1, X2, and X1*X2 will likely be masked by inflated standard errors, or multicollinearity [56,57]. Following Hainmueller, Mummolo [55] we relax the assumption of a linear effect. Fig 1 shows that the interactive effect of informality and access to a universal welfare state is in a negative direction, where the effect of informality on HALE reduces from low to medium values of the quality of a welfare state (see Fig 1).

**Fig 2:**
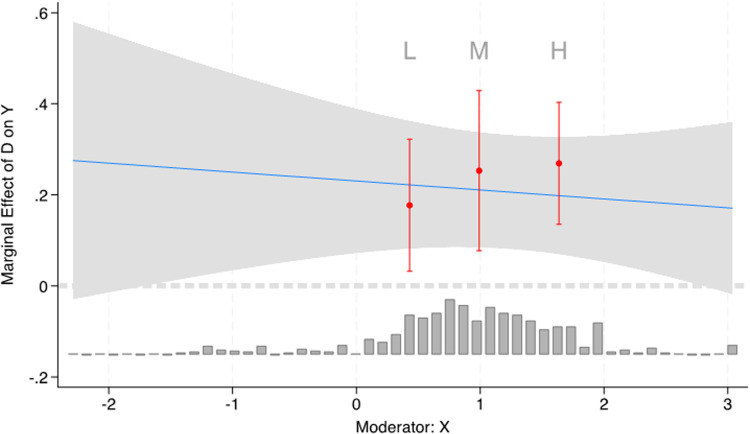
Margins plot of conditional effects of informality and access to welfare state on under 5 mortality rates.

In column 4, we test the conditional effect of informality and a universal welfare state on the under-five mortality rate (see Fig 2).

As seen there, again, increasing informality in a universal welfare state increases the under-five mortality rates (recall that it is positive for the independent term of informality). These non-linear effects do not suggest, in both cases, that access to universal welfare moderates the effects of the size of informality in a direction more favourable for population health standards.

Thus far, we have demonstrated that the conditional effect between the size of informality and greater universal access to welfare increases the under-five mortality rate and reduces HALE. We then move on to test the robustness of the association between the share of informality and one of our main dependent variables, namely, the under-five mortality rate to bias from selection on observables. Here, additional potential confounders were added to the basic model presented in Table 1, namely political corruption, public corruption, tax revenues as a share of GDP, and the degree of trade dependence. None of these had any effect on the outcomes, and informality´s effects retain statistical significance despite these alternative specifications of the model.

Next, we change the pooled model that has a common intercept for all units and estimate the random effects model where we allow the intercept to vary across units. We retain the OLS approach but compute standard errors robust to first-order serial correlation (temporal dependence) and general forms of cross-sectional dependence (spatial dependence) using the Driscoll-Kraay standard error method [58]. We use the Hausman test to assess between the fixed and random effects specifications. Accounting for heterogeneity in the units reduces the significance of results of our main variable for the full sample, but they remain highly significant and positive in both a fixed effects specification and random effects specification when we drop a handful of rich democracies from the sample (Western Europe, North America, Oceania plus Japan). Presumably, these countries show very little variation on under-five mortality rates and also potentially in terms of the main independent variable, informality. Variance factor inflation scores (VIF) do not suggest that are models suffer multicollinearity. Thus, there is good evidence in this sample of data to suggest that informality relates positively with poorer population health, independently of the controls, such as GDP per capita. Given that the basic models in Table 1 each explain more than 80% of the variance (R-square values > 0.81), we believe that the association between the exposure and the outcome is significant also in real-world terms. Our expectation that the size of informality on health would be moderated in a favourable direction by access to a quality welfare state is unsupported in the data.

## Discussion

This paper is the first to assess the association between the size of informality and population health on a global sample and to consider the role of macro-level factors measured as the quality of welfare state policies, which may moderate this relationship. Overall, the results across Table 1 are consistent with the view that a higher level of informality in a society is associated with worse population health outcomes, particularly when assessing the mother-child health status and equal access to health. Our examination of informality on HALE did not support the view that higher level of informality associates with higher overall health adjusted mortality. This is surprising since worse overall health and health inequalities are reported in many individual-level studies [8,9,59,60] and our expectation was that HALE as a measure might pick up these outcomes. As discussed earlier, comparing HALE across low-income and high-income country samples might yield different results [43]. We suspect that HALE, which mixes all diseases, is a less precise indicator of the typical disease burden of the poorest countries more prone to higher levels of informality. The use of HALE across these settings could partly explain this unexpected result although per capita GDP does indeed strongly capture the associationwith health across our models.

The association between informality and the narrower measures of health capturing mother-child health dynamics clearly links informality to worse population health. These statistically significant effects, which are also substantively large, are thus in accordance with the theory and in line with existing evidence at the population-level [16]. Our results, taken together, suggest that informality especially affects the health of women and children, because of the strong associations with maternal and child health indicators [12,22,60].

Poverty among women due to insecure employment conditions and ineligibility for social protections, such as paid leave for childcare, is likely an explanation for worse mother-child health [61]. For example, malnutrition and maternal care services are key determinants of both child mortality [62,63] and maternal mortality [64]. Studies that compare to formal workers, has found that informally working women are more likely to have undernourished children ([65,66] and that they are less likely to utilizing maternal health care services [67]. Informally working women’s lack of access to protections such as maternity leave, and their inadequate incomes could partly explain their worse health outcomes. As will be discussed later, our expectation that universal welfare states would thus condition the harmful effect of informality on these health outcomes, was however not supported. However, this may reflect the extreme vulnerability of many informal workers even in universal welfare states since eligibility to social security is still largely determined by formal employment [68].

Looking across the columns at the control variables, we see that per capita income level strongly correlates with better population health. Considering under-five mortality, as an example, higher levels of informality were linked to higher rates of under-five mortality. According to our findings, if per capita GDP would be increased by a standard deviation, for example, the under-five mortality rate would be reduced by roughly 65% of a standard deviation of the under-five mortality rate, which amounts to 15 fewer deaths per 1000 live births. These results imply that poorer countries could grow into better health, mutually reinforcing in virtuous cycles; i.e., higher incomes beget higher human capital and vice versa [48,69]. On the other hand, increased GDP has been seen to have less of an effect on under-five mortality in poor countries compared to rich countries [70], and because informality is more prevalent in low-income countries, it could, therefore be more appropriate to focus on improving the health of informal workers. Moreover, the policy tools in the hands of governments for reducing the effects of informality are likely more realistic than those designed to increase per capita incomes per se, for example, by regulating hiring and firing practices and other institutional changes that improve inclusivity in the formal work force. In our study, no other macro-level controls mattered much in predicting better population health. For example, higher levels of electoral democracy seem to matter little in predicting better population health, a finding consistent with others who report that democracy does not reduce child mortality [71].

The interactive effects are contrary to our expectations, and we found no evidence in this sample of data that access to high quality welfare state policies moderate the harmful effects of informal work on population health, after independent effects of income level measured as GDP per capita, for example, are held constant. This indicates that informality is linked to heath inequalities independently of access to welfare services. Our results that more universally designed welfare policies do not cushion health-harming effects of informality is similar to studies looking at the moderating role welfare state regimes for the relationship between informality and health comparing with informal and formal workers [16,27]. Here, it was found that while informal workers overall reported worse health compared to formal workers across all regime types, health inequalities were surprisingly smallest in the least generous welfare state regimes.

Our results possibly reflect two factors: firstly, that informal workers and their families do not have sufficient access to welfare policies, despite these being more universal on paper. There are ongoing discussions on how to best ensure that universal policies indeed truly reach informal workers, as such policies in reality are often restricted to those in formal employment [68,72]. This can lead to greater health inequalities between informal workers and others, and we may have picked up the heath consequence of this inequality of access; and secondly, that our result reflects reverse causality where countries with health problems invest more in welfare policies. Which of these issues form the mechanism should be pursued in future work addressing causality. In practice, however, financing universal policies in countries with large informal economies is complex [73].

Our analysis is only correlational and not causal, and we cannot be sure that informality ‘causes’ bad population health conditions measured in terms of mortality. Yet, there is a significant independent association between the size of informality and worse health outcomes even after GDPis accounted for. The associations allow us to infer that informality reduces population health standards, but this evidence is only true for child and maternal mortality and equal access to health, because informality weakly associates with HALE and in the opposite direction. These findings raise interesting questions about empirical testing of HALE as a comprehensive measure of population health when assessing the influence of poverty-related variables on health, HALE, which considers all diseases, is likely to mix those conditions that affect the rich differentially with those that affect the poor. To better understand HALE as a measure of population health, cause-specific HALE should be considered, and results should be stratified between LMICs and HICs as some already do [43].The main strength of this study, is that it covers a large number of countries, allowing for a broad overview of how informality is linked to population health. This allows us to detect spillover effects of informality, and to identify broad trends. However, available data on informal employment are generally scarce and potentially suffer biases. Informal employment per definition is not registered, thus complicating methods to measure it accurately. Indicators used by the ILO to produce data that we use for this study may not capture the true size of the informal workforce in some locations and would remain rough estimates. As data limitations will always be an issue when it comes to this unregistered phenomenon, future research should explore other more incisive and innovative ways of estimating the health consequences of informality.

## Conclusions

Informal workers dominate the workforces of many countries. While there is a great deal of theoretical discussion about the harmful effects of informality, the results have been mixed about whether and to what extent informality matters for population health standards. This study has found that larger sizes of informality, holding constant other broadly relevant confounders, negatively affect several mother-child health indicators and equal access to health. We did not find an apparent negative effect of informality on general HALE.

Moreover, there is conflicting evidence on whether access to a robust welfare system moderates the effects of informality on population health. Our results show that access to a quality welfare state does not necessarily reduce the adverse effects of informality on population health. To further understand the pathways between informality, health, and health inequalities, future studies should consider the interplay between informal work, macro-micro level factors, assessing the direction of causality more keenly. With informality being associated with worse maternal mortality and under-five mortality, attention to gendered effects of informality seems especially warranted.

## Supporting information

S1 Appendix. (DOCX)

S2 Appendix. (DOCX)
