# Supplementary material for: Informal employment, population health, and welfare policies: A global empirical analysis between 2011–2021
Source: PLoS One. 2025 Jun 26;20(6):e0325277. doi: 10.1371/journal.pone.0325277 (PMC12200695; doi:10.1371/journal.pone.0325277)
Supplement: S1 — (DOCX) [file pone.0325277.s001.docx]

**S1 APPENDIX**

Intercorrelation matrix

|  | 1 | 2 | 3 | 4 | 5 | 6 | 7 | 8 | 9 | 10 | 11 |
| --- | --- | --- | --- | --- | --- | --- | --- | --- | --- | --- | --- |
|  |  |  |  |  |  |  |  |  |  |  |  |
| 1) HALE | 1 |  |  |  |  |  |  |  |  |  |  |
| 2) Under 5 mortality | -0.8774 | 1 |  |  |  |  |  |  |  |  |  |
| 3) Maternal Mortality | -0.7807 | 0.8998 | 1 |  |  |  |  |  |  |  |  |
| 4) Mortality prenatal/nutrition | -0.7807 | 0.8137 | 0.7332 | 1 |  |  |  |  |  |  |  |
| 5) GDP per capita | 0.5534 | -0.419 | -0.3899 | -0.486 | 1 |  |  |  |  |  |  |
| 6) Total informal worker % | -0.5253 | 0.5427 | 0.496 | 0.6422 | -0.583 | 1 |  |  |  |  |  |
| 7) Female informal worker % | -0.5133 | 0.536 | 0.5181 | 0.6169 | -0.524 | 0.9658 | 1 |  |  |  |  |
| 8) Electoral democracy | 0.4851 | -0.542 | -0.3403 | -0.414 | 0.485 | -0.567 | -0.4899 | 1 |  |  |  |
| 9) Civil war ongoing | -0.2254 | 0.1302 | 0.2439 | 0.2148 | -0.17 | 0.2592 | 0.1926 | -0.1417 | 1 |  |  |
| 10) Peace exposure | 0.3799 | -0.442 | -0.4217 | -0.405 | 0.38 | -0.451 | -0.3855 | 0.3686 | -0.3947 | 1 |  |
| 11) Equal access to health | 0.6772 | -0.624 | -0.5899 | -0.73 | 0.61 | -0.7 | -0.6171 | 0.5685 | -0.2576 | 0.4387 | 1 |
| 12) Universal welfare | 0.3722 | -0.396 | -0.3435 | -0.408 | 0.335 | -0.477 | -0.4077 | 0.3996 | -0.2126 | 0.3106 | 0.603 |
