# Supplementary material for: Informal employment, population health, and welfare policies: A global empirical analysis between 2011–2021
Source: PLoS One. 2025 Jun 26;20(6):e0325277. doi: 10.1371/journal.pone.0325277 (PMC12200695; doi:10.1371/journal.pone.0325277)
Supplement: S2 — (DOCX) [file pone.0325277.s002.docx]

**S2 Appendix.**

Summary statistics

| Variable | Obs | Mean | Std. dev. | Min | Max |
| --- | --- | --- | --- | --- | --- |
|  |  |  |  |  |  |
| 1) HALE | 642 | 65.579 | 5.1376 | 45.242 | 72.33 |
| 2) Under 5 mortality | 642 | 18.957 | 23.435 | 2.1 | 118.3 |
| 3) Maternal Mortality | 480 | 78.404 | 142.32 | 2 | 812 |
| 4) Mortality prenatal/nutrition | 633 | 2.1555 | 0.9529 | 0.1894 | 4.207 |
| 5) GDP per capita | 642 | 17963 | 21738 | 328.11 | 1E+05 |
| 6) Total informal worker % | 642 | 15.638 | 12.241 | 0.6404 | 47.24 |
| 7) Female informal worker % | 642 | 6.6258 | 5.6616 | 0.2268 | 22.95 |
| 8) Electoral democracy | 642 | 0.6798 | 0.2121 | 0.124 | 0.926 |
| 9) Civil war ongoing | 642 | 0.0935 | 0.2913 | 0 | 1 |
| 10) Peace exposure | 642 | 38.768 | 21.369 | 0 | 59 |
| 11) Equal access to health | 642 | 1.1049 | 1.4701 | -2.264 | 3.31 |
| 12) Universal welfare | 642 | 0.972 | 0.7886 | -2.288 | 3.037 |
